# Synergistic Effects of Salicylic Acid and Melatonin on Modulating Ion Homeostasis in Salt-Stressed Wheat (*Triticum aestivum* L.) Plants by Enhancing Root H^+^-Pump Activity

**DOI:** 10.3390/plants11030416

**Published:** 2022-02-02

**Authors:** Neveen B. Talaat, Bahaa T. Shawky

**Affiliations:** 1 Department of Plant Physiology, Faculty of Agriculture, Cairo University, Giza 12613, Egypt; 2 Department of Microbial Chemistry, Biotechnology Research Institute, National Research Centre, Giza 12311, Egypt; btshawky44@yahoo.co.in

**Keywords:** antioxidant response, melatonin, nutrient uptake, root H^+^-pump activity, salicylic acid, salt stress, wheat (*Triticum aestivum* L.)

## Abstract

Salicylic acid (SA) and melatonin (MT) have been shown to play important roles in plant salt tolerance. However, the underlying mechanisms of SA–MT-interaction-mediated ionic homeostasis in salt-stressed plants are unknown. As a first investigation, this study aimed to clarify how SA–MT interaction affects H^+^-pump activity in maintaining the desired ion homeostasis under saline conditions and its relation to ROS metabolism. Wheat (*Triticum aestivum* L.) plants were grown under non-saline or saline conditions and were foliar sprayed with 75 mg L^−1^ SA or 70 μM MT. The SA+MT combined treatment significantly increased N, P, K^+^, Fe, Zn, and Cu acquisition, accompanied by significantly lower Na^+^ accumulation in salt-stressed plants compared to non-stressed ones. Additionally, it significantly enhanced ATP content and H^+^-pump activity of the roots. The mitigation was also detected in the reduced superoxide radical content, electrolyte leakage, and lipoxygenase activity, as well as increased superoxide dismutase, catalase, peroxidase, and polyphenol oxidase activities; K^+^/Na^+^, Ca^2+^/Na^+^, and Mg^2+^/Na^+^ ratios; relative water content; membrane stability index; and free amino acid accumulation in treated plants. The novel evidence shows that the higher root H^+^-pump activity in treated plants is a tolerance mechanism that increases the salt tolerance via maintaining ionic homeostasis.

## 1. Introduction

Soil salinization is one of the most damaging ecological stresses, which causes land desertification and the degradation of arable land. It affects about 20% of the irrigated land [1]. It is considered a complex abiotic stress in which ionic, osmotic, and oxidative stresses are involved [2,3,4]. Osmotic stress occurs shortly after exposure to salt stress and has detrimental effects on the plant ability to take up water and other nutrients [5,6]. Osmotic adjustment is one of the vital tolerance processes to osmotic stress, which can induce the synthesis of organic solutes [7]. Excessive accumulation of Na^+^ in the cytoplasm not only leads to K^+^ deficiency but also disrupts the protein biosynthesis, enzyme activity, and photosynthetic process [2,8]. The plant ability to maintain a low tissue Na^+^/K^+^ ratio has emerged as a significant salt tolerance feature [9,10]. Furthermore, in order to improve plant salt tolerance, harmful Na^+^ ions pumped into vacuoles must not leak back into the cytoplasm [11]. The electrochemical membrane proton gradient affects the removal of Na^+^ ions from the cell, which is catalyzed by the specific plasma membrane Na^+^/H^+^ exchanger [12]. In this regard, the plasma membrane H^+^-ATPase can act as a main transporter, pumping protons out of the cell to generate an electrochemical proton gradient, which helps in nutrient uptake, intracellular pH management, and plant salt adaptation [10]. In addition, salinity causes oxidative stress and increases the generation of ROS, which are highly cytotoxic and can react with essential biomolecules including lipids, proteins, and nucleic acids, resulting in lipid peroxidation, protein denaturation, and DNA mutation, respectively [3,6]. Enhancing the activity of plant antioxidant enzymes (superoxide dismutase, catalase, peroxidase, and enzymes involved in the ascorbate–glutathione cycle) is very important to counter oxidative stress [13,14,15].

Salicylic acid (SA, 2-hydroxybenzoic acid), as a plant growth regulator, can stimulate several biochemical events, resulting in a new metabolic state [7,16]. It has been shown that SA regulates plant salt tolerance by involving in redox homeostasis, suggesting that SA can interact with ROS signal pathways [17,18]. Several studies have shown that SA-regulated plant salt tolerance could contribute to maintaining cellular detoxification through the regulation of antioxidant enzyme activity, antioxidant molecules synthesis, organic solute accumulation, nutrient uptake, protein metabolism, and photosynthetic activity [7,16,19,20,21,22,23]. However, there is still a lack of information available regarding the core pathways precisely regulated by SA.

Melatonin (MT, *N*-acetyl-5-methoxytryptamine) is recognized as a novel metabolic regulator [9,22]. Moreover, MT can induce tolerance to salt stress, and this phenomenon has been reported in several plant species, such as rice [9], wheat [24], melon [25], naked oat [26], tomato [27], strawberry [28], and maize [29]. The evidence indicates that the exogenous application of MT enhances plant salt tolerance by scavenging ROS, improving photosynthetic efficiency, inducing nitrogen metabolism, and regulating stress-related transcription factors [22,23,30]. There is also evidence that MT maintains Na^+^/K^+^ homeostasis under salt stress through increasing both the root H^+^-pump activity and Na^+^/K^+^ transporter sensitivity to ROS and RNS [10]. Recently, Chen et al. [9] found that MT alleviates salt damage by decreasing the ROS level and increasing K^+^ retention in rice leaf mesophyll cells via regulation of *OsHAK* expression. Nevertheless, the regulation of MT-mediated ion homeostasis in salt-stressed plants is still not well understood.

Among the cereal crops, wheat (*Triticum aestivum* L.) is the most vital grain crop; however, it faces severe losses in its productivity due to salt stress [6]. Some reports have investigated the effects of separate application of SA or MT on plants exposed to salt stress; however, the underlying mechanisms of their combined treatment on plant salt tolerance are still unclear. To fill this gap, we conducted this investigation to evaluate the effect of SA and MT combined treatment on the nutrient uptake efficiency of salt-stressed wheat plants. Hence, we hypothesized that the co-application of SA and MT might improve wheat salt tolerance via maintaining ion homeostasis through the regulation of H^+^-pump activity and ATP content. To verify this hypothesis, the changes in nutrient acquisition (N, P, K^+^, Na^+^, Ca^2+^, Mg^2+^, Fe, Zn, Cu), mineral elements ratios (K^+^/Na^+^, Ca^2+^/Na^+^, Mg^2+^/Na^+^), ATP content, H^+^-pump activity, total free amino acid concentration, and enzyme activity (superoxide dismutase, catalase, peroxidase, polyphenol oxidase, lipoxygenase), along with certain other physiological responses such as relative water content, membrane stability index, electrolyte leakage, and superoxide radical content, were evaluated in wheat plants grown under both non-saline and saline conditions and foliar-sprayed with SA or MT. The results will contribute to further understanding the roles played by SA and MT in ameliorating salt stress.

## 2. Results

### 2.1. Foliar Applications of SA and MT Alleviate the Growth and Productivity Reduction Induced by Salt Stress

Wheat-growth- and yield-related traits, in terms of the total leaf area, shoot dry weight, root dry weight, number of grains, and grain yield, displayed considerable reductions in response to the increasing levels of salt in comparison to those of the control (non-stressed) plants (Table S1). On the contrary, foliar applications of SA or MT alleviated the salt-stress-induced reductions in these parameters, whilethe dual application (75 mg L^−1^ SA + 70 μM MT) appeared to be much more effective in alleviating the deleterious effects of saline conditions (Table 1).

### 2.2. Spraying of SA and MT Regulates Nutrient Uptake Efficiency in Salt-Stressed Plants

**Figure 1:**
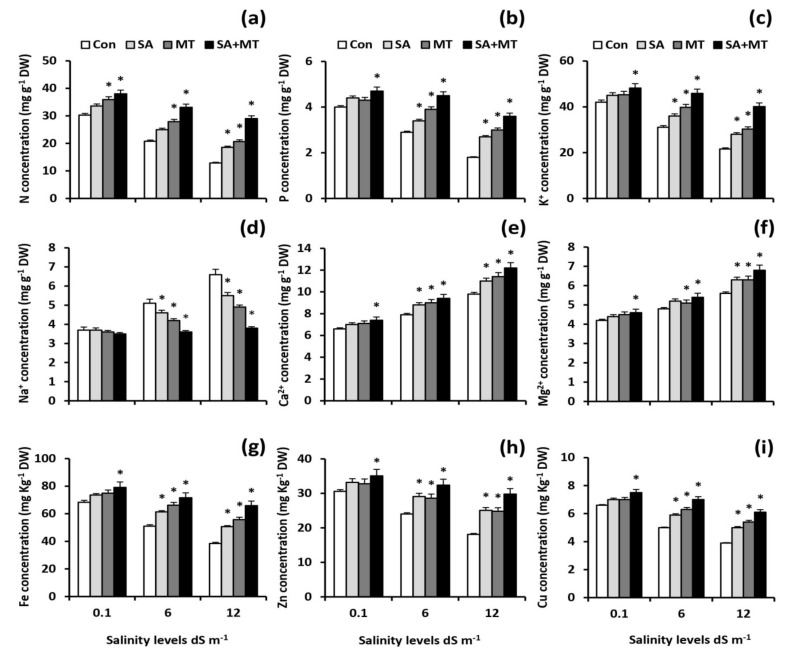
Influence of salicylic acid (SA, 75 mg L^−1^), melatonin (MT, 70 μM), and SA (75 mg L^−1^) + MT (70 μM) foliar application treatments on the concentrations of (**a**) nitrogen (N), (**b**) phosphorus (P), (**c**) potassium (K^+^), (**d**) sodium (Na^+^), (**e**) calcium (Ca^2+^), (**f**) magnesium (Mg^2+^), (**g**) iron (Fe), (**h**) zinc (Zn), and (**i**) copper (Cu) (mg g^−1^ DW) in shoots of wheat plants grown under 0.1, 6, and 12dS m^−1^ salinity levels. Data are means of four replicates (n = 4) and bars show standard errors (±SE). Asterisk represent significant differences between treatments at the *p* < 0.05 level according to LSD test.

**Figure 2:**
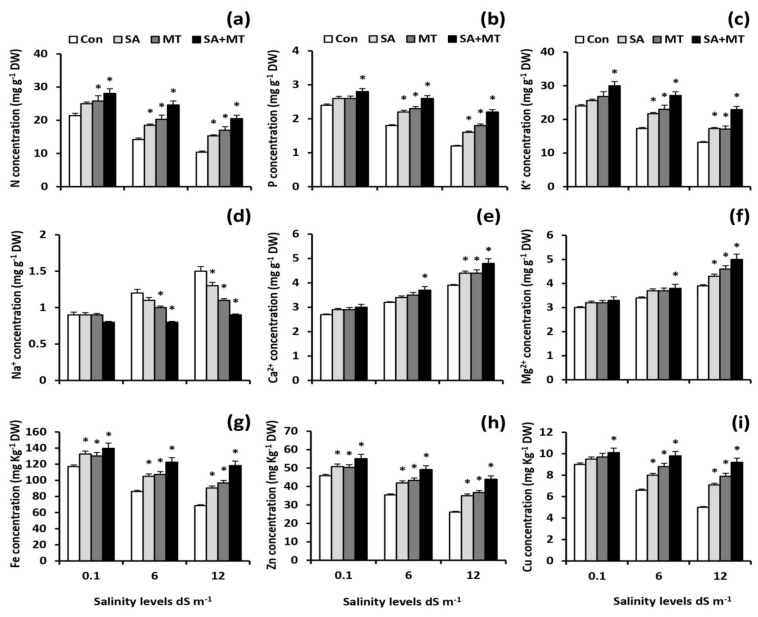
Influence of salicylic acid (SA, 75 mg L^−1^), melatonin (MT, 70 μM), and SA (75 mg L^−1^) + MT (70 μM) foliar application treatments on the concentrations of (**a**) nitrogen (N), (**b**) phosphorus (P), (**c**) potassium (K^+^), (**d**) sodium (Na^+^), (**e**) calcium (Ca^2+^), (**f**) magnesium (Mg^2+^), (**g**) iron (Fe), (**h**) zinc (Zn), and (**i**) copper (Cu) (mg g^−1^ DW) in grains of wheat plants grown under 0.1, 6, and 12dS m^−1^ salinity levels. Data are means of four replicates (n = 4) and bars show standard errors (±SE). Asterisk represent significant differences between treatments at the *p* < 0.05 level according to LSD test.

To elucidate the mechanism underlying how SA and MT treatments modulate nutrient acquisition, the concentrations of different ions in shoots and grains of wheat plants were assayed. The results showed that soil salinization was associated with considerable reductions in the concentrations of N, P, K^+^, Fe, Zn, and Cu, as well as considerable increases in Na^+^, Ca^2+^, and Mg^+2^ levels. Interestingly, stressed MT- and SA-treated plants showed significant lower Na^+^ accumulation along with higher N, P, K^+^, Fe, Zn, and Cu acquisition when compared with untreated stressed plants (Figure 1a–i and Figure 2a–i). The best response was registered with SA and MT combined treatment. Thistreatmentsignificantly (*p* < 0.05) elevated the N levels in grains of treated plants by 31.3%, 73.2%, and 97.1%; the P levels by 16.7%, 44.4%, and 83.3%; the K^+^ levels by 25.0%, 56.6%, and 73.5%; the Fe levels by 19.2%, 42.2%, and 72.4%; the Zn levels by 20.3%, 39.0%, and 68.2%; and the Cu levels by 12.2%, 48.5%, and 84.0% at 0.1, 6.0, and 12.0 dS m^−1^ salinity levels, respectively, compared with control treatment. In addition, the combined treatment significantly decreased Na^+^ values by 33.3 % and 40.0 % in grains of treated plants compared to the values of untreated plants at 6.0 and 12.0 dS m^−1^ salinity levels, respectively.

### 2.3. Exogenously Applied SA and MT Maintain K^+^/Na^+^, Ca^2+^/Na^+^, and Mg^2+^/Na^+^ Homeostasis under Saline Conditions

**Figure 3:**
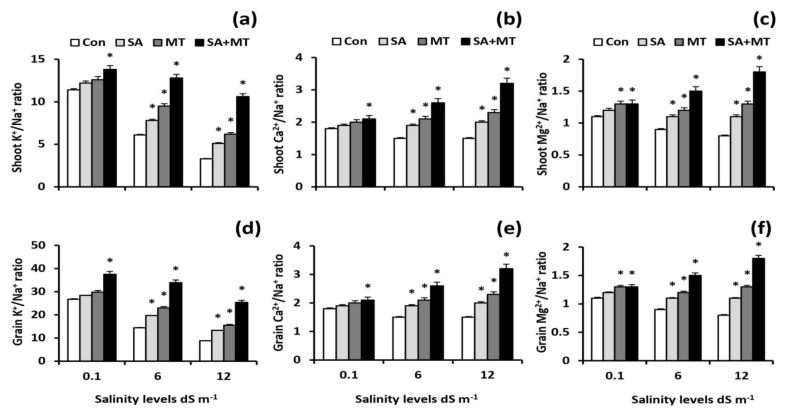
Influence of salicylic acid (SA, 75 mg L^−1^), melatonin (MT, 70 μM), and SA (75 mg L^−1^) + MT (70 μM) foliar application treatments on the ratios of (**a**) K^+^/Na^+^, (**b**) Ca^2+^/Na^+^, and (**c**) Mg^2+^/Na^+^ in shoots, as well as (**d**) K^+^/Na^+^, (**e**) Ca^2+^/Na^+^, and (**f**) Mg^2+^/Na^+^ in grains of wheat plants grown under 0.1, 6, and 12 dS m^−1^ salinity levels. Data are means of four replicates (n = 4) and bars show standard errors (±SE). Asterisk represent significant differences between treatments at the *p* < 0.05 level according to LSD test.

To explicate how SA and MT treatments eliminate the adverse effects of salt stress, the K^+^/Na^+^, Ca^2+^/Na^+^, and Mg^2+^/Na^+^ ratios were quantified. The results pointed out that these ratios were considerably decreased in shoots and grains of plants grown under saline conditions, whereas they were significantly increased in salt-stressed plants treated with SA or MT (Figure 3a–f). The greatest ratios were obtained by SA and MT combined treatment.This treatment significantly (*p* < 0.05) improved the K^+^/Na^+^ ratios in grains of treated plants by 40.4%, 135.4%, and 188.6%; the Ca^2+^/Na^+^ ratios by 26.7%, 70.3%, and 103.8%; and the Mg^2+^/Na^+^ ratios by 24.2%, 71.4%, and 115.4% compared to the values for untreated plants at 0.1, 6.0, and 12.0 dS m^−1^ salinity levels, respectively.

### 2.4. Foliar Applications of SA and MT Ameliorate ATP Content and H^+^-Pump Activity in Wheat Roots under Salinity Conditions

**Figure 4:**
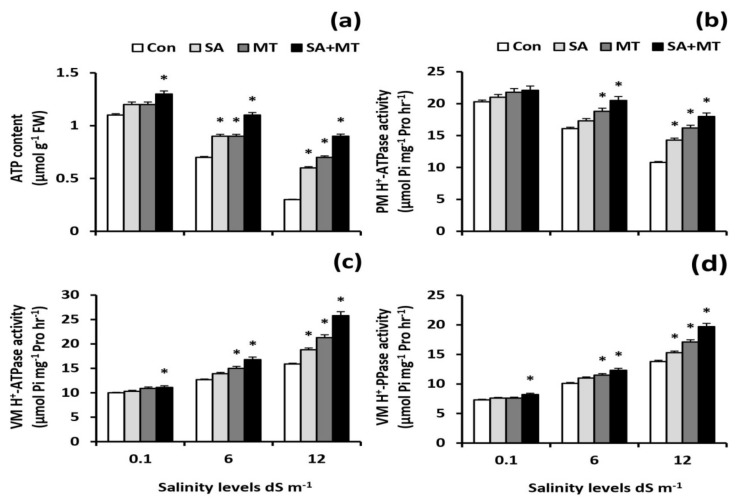
Influence of salicylic acid (SA, 75 mg L^−1^), melatonin (MT, 70 μM), and SA (75 mg L^−1^) + MT (70 μM) foliar application treatments on the (**a**) ATP content, (**b**) plasma membrane (PM) H^+^ -ATPase activity, (**c**) vacuole membrane (VM) H^+^ -ATPase activity, and (**d**) vacuole membrane (VM) H^+^-PPase activity in roots of wheat plants grown under 0.1, 6, and 12dS m^−1^ salinity levels. Data are means of four replicates (n = 4) and bars show standard errors (±SE). Asterisk represent significant differences between treatments at the *p* < 0.05 level according to LSD test.

As shown in Figure 4a, salt stress considerably decreased the ATP content in wheat roots, whereas SA and MT foliar applications alleviated the decreasing effect caused by salt stress and significantly increased its content. The SA and MT combined treatment appeared to be much more effective in alleviating this detrimental effect of salt stress and significantly increased the ATP content levels by 18.2%, 57.1%, and 200.0% under 0.1, 6.0, and 12.0 dS m^−1^ salinity levels, respectively, when compared with untreated plants.

It is well known that PM H^+^-ATPase plays an important role in ion transport and that its activity is down-regulated under salt stress. However, SA and MT applications significantly increased its activity (Figure 4b). Interestingly, the VM H^+^-ATPase and VM H^+^-PPase activities were increased under salt stress; moreover, they were further increased by SA and MT treatments (Figure 4c,d). The maximum activities were detected by SA and MT co-application, which significantly (*p* < 0.05) enhanced the PM H^+^-ATPase activity levels by 27.3% and 66.7%, the VM H^+^-ATPase activity levels by 32.3% and 62.3%, and the VM H^+^-PPase activity levels by 21.8% and 42.8% at 6.0 and 12.0 dS m^−1^ salinity levels, respectively, when compared with untreated plants.

### 2.5. SA and MT Treatments Improve Leaf Relative Water Content (RWC) and Total Free Amino Acid Concentration Levels

In view of the effects of salt treatments on leaf RWC, it was postulated that salt treatments considerably reduced its value. However, exogenous SA and MT applications were associated with significant increases in its levels (Table 2). The most noteworthy defensive impact was achieved by the SA and MT combined treatment, whichsignificantly (*p* < 0.05) improved the RWC levels by 13.6%, 38.0%, and 61.5% compared to the values for control plants at 0.1, 6.0, and 12.0 dS m^−1^ salinity levels, respectively. In addition, the total free amino acid concentration was sharply increased in leaves of salt-stressed plants; moreover it was further increased by SA and MT treatments (Table 2). The maximum values were detected with SA and MT co-application. This significantly enhanced the total free amino acid content levels by 42.1% and 63.3% compared to values for control plants at 6.0 and 12.0 dS m^−1^ salinity levels, respectively.

### 2.6. Exogenous Treatments of SA and MT Prevent ROS Accumulation under Saline Conditions

One of the severe outputs of saline conditions is the O_2_^•−^ generation in excessive amounts in wheat leaves. Conversely, SA and MT applications significantly lowered its values in salt-stressed plants (Table 2). The maximum ameliorative effect was detected with SA and MT co-application, which significantly (*p* < 0.05) reduced the O_2_^•−^ content levels by 25.7% and 31.8% compared to the values for control plants at 6.0 and 12.0 dS m^−1^ salinity levels, respectively.

### 2.7. Exogenously Applied SA and MT Protect Leaf Cell Membrane Integrity

**Figure 5:**
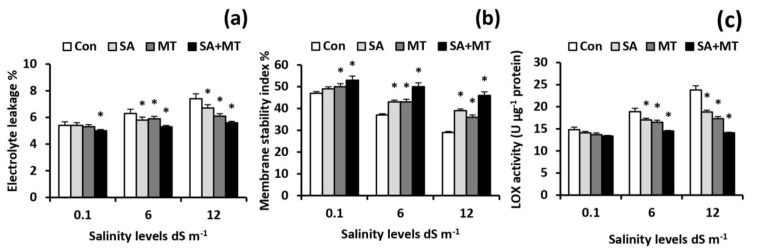
Influence of salicylic acid (SA, 75 mg L^−1^), melatonin (MT, 70 μM), and SA (75 mg L^−1^) + MT (70 μM) foliar application treatments on the (**a**) electrolyte leakage (%), (**b**) membrane stability index(%), and (**c**) lipoxygenase (LOX) activity levels in leaves of wheat plants grown under 0.1, 6, and 12 dS m^−1^ salinity levels. Data are means of four replicates (n = 4) and bars show standard errors (±SE). Asterisk represent significant differences between treatments at the *p* < 0.05 level according to LSD test.

Alterations of the cellular membrane were assessed by determining electrolyte leakage (EL) and membrane stability index (MSI) values, as well as lipoxygenase (LOX) activity. As shown in Figure 5a,c, the EL value and LOX activity were increased in response to saline treatments, whereas these increases were significantly attenuated by the exogenous SA and MT applications. The maximum ameliorative effect was detected with SA and MT co-application, which significantly (*p* < 0.05) reduced the EL levels by 15.9% and 24.3% and the LOX activity levels by 23.3% and 40.8% compared to the values for control plants at 6.0 and 12.0 dS m^−1^ salinity levels, respectively. On the contrary, salt treatments considerably reduced the MSI values; however, exogenous SA and MT applications were associated with significant increases in MSI values (Figure 5b). The highest protective effect was achieved by SA and MT combined treatment. MSI values were enhanced in treated plants by 12.8%, 35.1%, and 58.6%, at 0.1, 6.0, and 12.0 dS m^−1^ salinity levels, respectively, when compared with untreated ones.

### 2.8. SA and MT Foliar Treatments Improve the Antioxidant Activity under Saline Conditions

**Figure 6:**
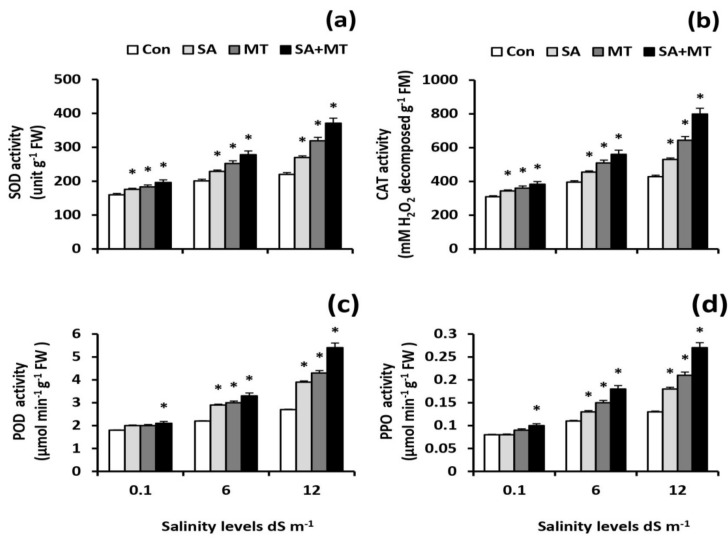
Influence of salicylic acid (SA, 75 mg L^−1^), melatonin (MT, 70 μM), and SA (75 mg L^−1^) + MT (70 μM) foliar application treatments on the activitylevels of (**a**) superoxide dismutase (SOD), (**b**) catalase (CAT), (**c**) peroxidase (POD), and (**d**) polyphenol oxidase (PPO) in leaves of wheat plants grown under 0.1, 6, and 12 dS m^−1^ salinity levels. Data are means of four replicates (n = 4) and bars show standard errors (±SE). Asterisk represent significant differences between treatments at the *p* < 0.05 level according to LSD test.

Soil salinization increased the activity levels of SOD, CAT, POD, and PPO in wheat leaves; moreover, their activity levels were further increased by SA and MT treatments (Figure 6a–d). Co-application of SA and MT yielded the best response and significantly (*p* < 0.05) increased the SOD activity levels by 22.5%, 38.3%, and 68.6%; the CAT activity levels by 23.9%, 41.7%, and 86.7%; the POD activity levels by 16.7%, 50.0%, and 100.0%; and the PPO activity levels by 25.0%, 63.6%, and 107.7% compared to the values of control plants at 0.1, 6.0, and 12.0 dS m^−1^ salinity levels, respectively.

## 3. Discussion

Salt stress is an important environmental constraint that can affect the growth and productivity of wheat [3,6]. Previous studies have shown that the exogenous application of SA or MT enhances plant salt tolerance [7,9,10,16]. However, our knowledge regarding the mechanisms involved in their combined treatment-mediated salt tolerance still remains fragmentary. In the present study, as a first investigation we examined the effects of exogenously applied SA and MT on the nutrient uptake efficiency in wheat plants grown in salty soils. Our results clearly showed that co-application of SA and MT can alleviate the deleterious impacts of salt stress on wheat production by maintaining ion homeostasis through the regulation of H^+^-pump activity and ATP content.

**Figure 7:**
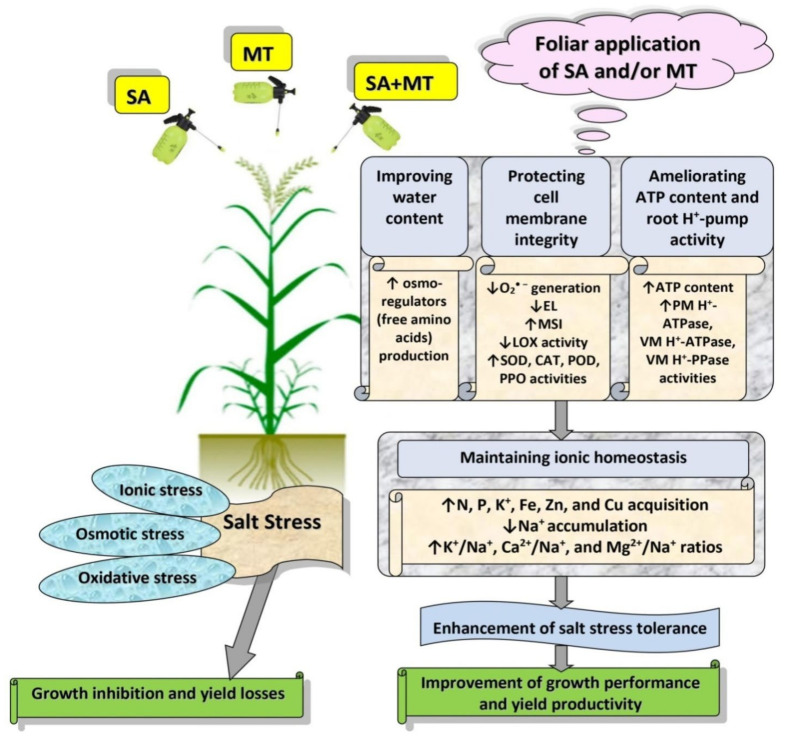
Foliar applications of SA and MT alleviate salt stress impacts on wheat growth and productivity by improving the ATP content, root H^+^-pump activity, water content, and ROS detoxification, which in turn maintain the ionic homeostasis.

In the present study, salt stress severely impaired wheat growth and productivity (Table 1) by interfering with essential nutrient uptake (Figure 1a–i and Figure 2a–i); negatively affecting water content (Table 2), electrolyte leakage (Figure 5a), and membrane stability index (Figure 5b); and inducing oxidative stress (Table 2 and Figure 6a–d). On the contrary, in agreement with previous studies [9,10,19,21,26,27], we observed that exogenous applications of SA and MT ameliorate salt-induced reductions in wheat production by regulating ions uptake, increasing the preferential accumulation or exclusion of inorganic ions (Figure 1a–i and Figure 2a–i), altering the biological membrane structure (Figure 5a–c), reducing oxidative damage, suppressing O_2_^•−^ production (Table 2), and enhancing the antioxidant enzyme activity (Figure 6a–c). These findings reveal that SA and MT can act as active growth regulators involved in wheat salt tolerance by maintaining optimal mineral nutrition and blocking ROS burst (Figure 7).

In the current study, salt stress significantly affected plant nutrient acquisition, whereas SA and MT exogenous applications alleviated the salt-induced disturbance in ionic status. Our results showed that SA and MT improve the ability of stressed plants to absorb N, P, K^+^, Fe, Zn, and Cu (Figure 1a–c,g–i) and exclude Na^+^ (Figure 1d). These findings can be attributed to the maintenance of membrane properties [9,10] (Figure 5a–c) by SA and MT through decreasing ROS burst and improving antioxidant enzyme activity (Figure 6a–c). Strong evidence has demonstrated that SA and MT elevate the nutrient acquisition levels by rectifying the damage in plasma membranes [9,10,19]. Moreover, in the present investigation, we found that the protective effects of SA and MT on nutrient uptake could be related to the higher H^+^-ATPase activity (Figure 4b,c). This is in line with previous findings [9,31,32], which showed that the H^+^-ATPase activity is closely related to the mineral status in stressed plants. Our findings suggest that SA and MT can mitigate the growth and yield reductions by altering the ionic status within the plant in favor of salt stress tolerance.

It is well documented that plant salt tolerance is closely related to the ability to maintain adequate K^+^/Na^+^, Ca^2+^/Na^+^, and Mg^2+^/Na^+^ ratios [13,33]. Consistent with these reports, our study showed significant increases in K^+^/Na^+^, Ca^2+^/Na^+^, and Mg^2+^/Na^+^ ratios in salt-stressed treated plants (Figure 3a–f). Under salt stress conditions, Na^+^ enters the cytosol and causes plasma membrane depolarization, resulting in the continuous efflux of K^+^, which can lead to an increase in the Na^+^/K^+^ ratio in the cytosol [2]. Previous studies showed that MT promotes the influx of K^+^ and improves K^+^/Na^+^ homeostasis by regulating K^+^/Na^+^ transporters [9,10]. Maintaining a high selectivity for K^+^ ion in spite of an excess of Na^+^ ions may support the ability of stressed treated plants to counteract salt injuries. Overall, our results clearly confirm that SA and MT play important roles in alleviating salt damage by maintaining ion homeostasis.

In order to maintain low Na^+^/K^+^, Na^+^/Ca^2+^, and Na^+^/Mg^2+^ ratios under saline conditions, an accumulation of excessive amounts of Na^+^ in the cytosol should be prevented, which can be achieved by maintaining the H^+^-pump activity [10,18]. In the present study, significant increments in root ATP content and root H^+^-pump activity were detected via the applications of SA and MT under saline conditions (Figure 4a–d), supporting the concept that SA and MT applications are conducive to maintaining the plasma membrane polarization and improving the cytosolic ion homeostasis. An increase in root H^+^-pump activity could increase Na^+^ exclusion and increase K^+^ uptake [12]. Strong evidence has demonstrated that MT increases the root H^+^-pump activity, thereby promoting Na^+^ efflux and K^+^ influx as well as maintaining the K^+^/Na^+^ ratio [34]. The observed increases in H^+^-ATPase activity caused by SA and MT could be attributed to the fact that SA and MT may operate directly as an antioxidant to scavenge ROS or indirectly by triggering antioxidant responses [9,19,21] (Table 2 and Figure 6a–c). Consistent with this suggestion, Yan et al. [10] showed an enhancement in H^+^-pump activity in rice MT-treated plants under conditions of decreasing ROS content. The present study confirmed that the higher root H^+^-pump activity in SA- and MT-treated plants is a tolerance mechanism that increases the salt tolerance via maintaining ionic homeostasis.

The maintenance of water status is the most intrinsic determinant of plant adaptations to salt stress [33]. Our results indicated a drastic decline in leaf RWC with an increase in salinity level (Table 2). The reduction in leaf RWC in salt-stressed wheat plants might be associated with salt-induced water imbalance and decreased osmotic potential. Our results also revealed that SA and MT combined treatment restores the RWC near to control level at 6.0 and 12.0 dS m^−1^ salinity levels. The positive effects of combined treatment on RWC could be due to its stimulatory effects on osmoregulator (free amino acid) production (Table 2) and lower accumulation of Na^+^ under saline conditions, as was also reported by [21,25].

Osmoprotectants such as amino acids participate in regulating osmotic pressure in the cytoplasm and stabilizing proteins and membranes when plants are grown under saline conditions [13]. Our results demonstrated increased accumulation of these osmolytes with the increase in salinity level (Table 2), perhaps due to water stress generated by salinity, as evident from the decreased RWC in wheat leaves. Our findings also revealed that the total free amino acid concentration was further increased by both SA and MT. This accumulation may play a role in osmotic adaptation. Our findings suggest that SA and MT protect wheat plants from salt stress by regulating osmotic potential, as evident from the relatively high leaf water contents.

One of the major injuries due to salinity stress is the production of ROS-like O_2_^•−^. Our results showed that salt-stress-induced accumulation of O_2_^•−^ was suppressed by SA and MT applications (Table 2). Several studies have demonstrated that SA can operate directly as an antioxidant to scavenge the ROS or indirectly by regulating redox balance [18,19,21], thereby alleviating salt stress injury. Likewise, MT serves as an effective endogenous-free radical scavenger that directly removes ROS or as an antioxidant to regulate the transcription levels of genes related to the antioxidant system [9,26,30]. Other reports revealed that SA and MT might exert their roles in maintaining ion homeostasis by regulating ROS production [9,10,19]. Therefore, it seems reasonable to speculate that SA and MT enhance wheat salt tolerance, possibly through the regulation of ROS burst.

Plants usually produce high ROS under saline conditions, which consequently causes oxidative damage and impaired membrane lipid functions [3,6]. This is consistent with our results, as we showed a positive correlation between increasing salinity levels and the extent of plasma membrane damage. On the contrary, SA and MT applications under salinity conditions stabilized membranes and significantly decreased the solute leakage (Figure 5a,b), indicating that these treatments may be able to repair the disrupted cellular membrane and reduce salt-induced oxidative damage. This finding is in line with the broad spectrum antioxidative properties of SA and MT, which are capable of directly scavenging ROS [18,30]. Furthermore, as shown in Figure 5c, higher ROS production in salt-stressed plants increased LOX activity, whereas SA and MT treatments diminished oxidative injuries via decreasing oxidative activity. These results corroborate the findings of [26].

Plants have developed defensive mechanisms that consist of antioxidants with enzymatic or non-enzymatic activity to cope with oxidative damage and to reduce excessive ROS accumulation [3,6]. Several studies have demonstrated that SA and MT increase the activity of enzymatic antioxidants under salt stress [9,19,21,25,26]. Consistently, our results confirmed these previous works. As shown in Figure 6a–d, SA and MT increased SOD, CAT, POD and PPO activity levels in salt-stressed wheat plants, leading to scavenging of excessive ROS and reducing oxidative damage. Overall, exogenously applied SA and MT promote the enzymatic antioxidant defense system that facilitates prompt removal of excess ROS, thereby enhancing wheat salt tolerance.

## 4. Materials and Methods

### 4.1. Plant Materials and Experimental Design

A pot experiment was conducted in the greenhouse of the Plant Physiology Department, Faculty of Agriculture, Cairo University, Egypt, under natural light and temperature conditions, with average day/night temperature conditions of 22/16 ± 2 °C and average humidity of 65%. The experiment was repeated twice, on September 10 of 2019 and 2020. Wheat (*Triticum aestivum* L. cv. Sids 14) grains were obtained from the Wheat Research Department, Agriculture Research Center, Ministry of Agriculture, Egypt. Sids 14 cultivar was selected based on its high yield productivity. Its salt tolerance was increased by using SA and MT foliar applications.

The pots were 30 cm in diameter and 35 cm in height and contained 15 kg of clay loamy soil (sand 37%, silt 28%, clay 35%). Ammonium nitrate (33.5% N), calcium superphosphate (15.5% P_2_O_5_), and potassium sulfate (48% K_2_O) were applied at rates of 2.0, 2.0, and 0.5 g pot^−1^, respectively. In addition, 2.0 g pot^−1^ ammonium nitrate was added 30 days after planting. The soil chemical analysis was carried out following the procedures in [35] and as presented in Table S2.

Before sowing, pots were divided into three groups. The first one was assigned as control (non-saline; 0.1 dS m^−1^) and the other two groups were assigned to two levels of salinity treatment (6.0 and 12.0 dS m^−1^ salinity levels, obtained by adding to the soil a mixture of NaCl, CaCl_2_, and MgSO_4_ at a molar ratio of 2:2:1, respectively).

The wheat plants at 45 and 90days old at each salinity level (0.1 dS m^−1^ (non-saline), 6.0, and 12.0 dS m^−1^) were foliar sprayed with 0.00 distilled water (DW), 75 mg L^−1^ SA, 70 μM MT, and 75 mg L^−1^ SA + 70 μM MT. The concentrations of 75 mg L^−1^ SA and 70 μM MT were the most effective concentrations according to preliminary experiments within a range of concentrations ranging from 0 to 100 μM for MT and from 0 to 100 mg L^−1^ for SA. SA and MT were purchased from Sigma-Aldrich (St. Louis, MO, USA) and were dissolved in a sufficient quantity of ethanol. Tween-20 (0.05%) was added as a surfactant at the time of treatment. During the two growing seasons, controlled irrigation was applied to each pot at the reference crop evapotranspiration (ET0) values.

The experimental layout involved a completely randomized design with two factors: three levels of salinity (0.1 dSm^−1^ (non-saline), 6.0, and 12.0 dS m^−1^) and four spraying treatments (0.00 distilled water (DW), 75 mg L^−1^ SA, 70 μM MT, and 75 mg L^−1^ SA + 70 μM MT). Each treatment included four replicates and each replicate included six plants gathered from the same pot.

### 4.2. Plant Growth and Plant Productivity Measurements

The 70-day-old plants were sampled (after 25 days of SA or MT first applications) to measure total leaf area, shoot dry weight, and root dry weight values per plant. The total leaf area plant^−1^ was estimated using a portable leaf area meter (LI-COR 3000, Lambda Instruments Corporation, Lincoln, NE, USA). Shoot and root dry weights per plant^−1^ were measured following oven drying at 70 °C for 48 h. Each treatment included four replicates and each replicate included six plants gathered from the same pot. At maturity, the number of grains and grain yield per plant were recorded.

The plants were sampled at 70 days of age (after 25 days of SA and MT first applications) to assess the following physiological and biochemicalparameters.

### 4.3. Determination of Mineral Elements

Dried ground shoots and grains (0.5 g) were digested in a mixture of boiling perchloric acid and hydrogen peroxide for 8 h until a transparent solution was obtained. Nitrogen concentration was obtained using the modified micro-Kjeldahl method following [36]. The phosphorus concentration was assessed using the vanadomolybdophosphoric method following [37]. Potassium and sodium concentrations were analyzed usinga flame photometer (ELE UK). Elemental analyses of calcium, magnesium, iron, zinc, and copper were determined using an atomic absorption spectrophotometer (Unicam 989-AA Spectrometer-UK).

### 4.4. Determination of ATP Content

ATP was extracted as described previously by [38]. ATP content was determined using an ATP Colorimetric/Fluorometric Assay Kit (BioVision, Milpitas, CA, USA) according to the manufacturer’s instructions.

### 4.5. Separation of the Plasma Membrane (PM) and Vacuole Membrane (VM) and Determination of H^+^-Pump Activity

Wheat roots about 2 cm from the tip were cut and washed with deionized water. Plasma and vacuole membranes were isolated according to the method used by [10]. The excised roots (10 g) were thoroughly chopped and homogenized (1/3, *w/v*) in a cold grinding medium containing the following: Hepes-Tris 60 mM, pH 7.5, source 300 mM, EDTA 5 mM, EGTA 0.5 mM, DTT 2 mM, 1.5% PVP, PMSF 2 mM, DTT 2 mM, BSA 0.1%. The homogenate was filtered through four layers of cheese cloth and centrifuged at 13,000× *g* for 20 min. The resulting supernatant was placed in a discontinuous sucrose gradient (containing 45%, 33%, and 15% (*m*/*v*) sucrose solution) and centrifuged at 80,000× *g* for 30 min. Then, 5 mL centrifuged sediment was taken at the interface between the 15–33% and 33–45% gradients. The 15–33% gradient was diluted to twice the volume with gradient centrifugation buffer (HEPES tris 20 mM, pH 7.5, EDTA 5 mM, EGTA 0.5 mM), while the 33–45% gradient was added to three to four times the volume of gradient centrifugation buffer. After shaking, mixing, and centrifugation at 100,000× *g* for 1 h, the supernatant was discarded. The precipitates were suspended with 0.5 mL suspension (HEPES tris 20 mM, pH 7.5, sucrose 300 mM, EGTA 0.5 mM, MgCl_2_⋅6H_2_O 0.5 mM) to obtain the VM and PM microcapsules, respectively. Protein concentrations were determined using the Coomassie brilliant blue method, using BSA as a standard.

H^+^-ATPase and H^+^-PPase activity levels were determined following the method used by [39]. Briefly, 15–20 μL tonoplast vesicles was added into 400 μL of the reaction medium, containing 30 mM Hepes–Tris (pH 6.0, pH 8.5 for H^+^-PPase assay), 3.0 mM MgSO_4_, 0.5 mM NaN_3_, 0.1 mM Na_3_VO_4_, 50 mM KCl, 0.1 mM ammonium molybdate, and 3.0 mM ATP (or 2.0 mM Na_4_PPi for H^+^-PPase assay). After the reaction at 37 °C for 20 min, 50 μL of TCA was added to stop the reaction. Inorganic phosphate released from ATP or PP hydrolysis was determined following the procedure used by [40].

### 4.6. Determination of Relative Water Content (RWC) and Total Free Amino Acid Concentration

The procedure described by [41] was followed to assess the RWC, which was determined in fresh leaf disks measuring 2 cm^2^ in diameter. Disks were weighed quickly and then immediately floated on DDW in Petri dishes to saturate them with water for the next 24 h in the dark. The adhering water on the disks was blotted and the turgor mass was noted. Dry mass weights of the disks were recorded after dehydrating them at 70 °C for 48 h. The following formula was used to measure the RWC%:

RWC = (fresh mass − dry mass)/(turgor mass − dry mass) × 100(1)

Total free amino acids were determined using the ninhydrin reagent method [42]. Here, 1 mL acetate buffer (pH = 5.4) and l mL chromogenic agent were added to 1 mL free amino acid extraction. The mixture was heated in a boiling water bath for 15 min. After cooling in tap water, 3 mL ethanol (60%, *v*/*v*) was added. The absorbance at 570 nm was then monitored.

### 4.7. Determination of Superoxide Radicals (O_2_^•−^)

To measure O_2_^•−^, 0.1 g fresh wheat leaves was homogenized in 900 µL buffer and centrifuged at 4000 rpm/min and 25 °C for 10 min. Then, 50 µL of the supernatant was mixed with 4 mL of reagent solution. The mixture was bathed at 37 °C for 40 min and then 2 mL reagent solution was added. An O_2_^•−^ detection kit (A052,Nanjing Jiancheng Bioengineering Inc., Nanjing, China) was used to assay the O_2_^•−^ content, as mentioned by [26]. The O_2_^•−^ content was recorded at a wavelength of 550 nm and was expressed as an increase in absorbance per dry weight.

### 4.8. Estimation of Electrolyte Leakage (EL) and Membrane Stability Index (MSI)

To access the leakage of ions from membranes, leaves were collected and washed with distilled water. They were placed in test tubes containing10 mL distilled water and kept in a water bath at 40 °C for 30 min while the electrical conductivity (C1) was recorded. Later, the same samples were placed in a water bath for 10 min at 100 °C and the electrical conductivity (C2) was noted. The electrolyte leakage was calculated using the formula used in [43]:

EL = [C1/C2] × 100(2)

The MSI was determined according to the method used by [44]. Wheat leaf samples (0.2 g) were incubated in 10 mL of deionized water and shaken gently for 24 h at room temperature. After incubation, the conductance was measured and noted as C1. Subsequently, leaf samples were further incubated at 120 °C for 20 min and then shaken for 24 h at room temperature. The final conductance was measured and noted as C2. The leaf MSI was calculated using the following formula:

MSI = [1 − C1/C2] × 100(3)

### 4.9. Enzyme Extraction and Assay

Wheat fresh leaves (0.5 g) were homogenized in 5 mL of ice-cold 100 mM phosphate buffer (pH 7.4) containing 1% polyvinyl pyrrolidine and 1 mM EDTA and then centrifuged at 15,000× *g* for 10 min at 25 °C. The supernatant was collected and used for the assays. The activity of superoxide dismutase (SOD, EC 1.15.1.1) was determined by monitoring its inhibition of the photochemical reduction of nitro blue tetrazolium as described by [45]. One unit of enzyme activity was defined as the amount of enzyme bringing about a 50% inhibition ofthe reduction rate of nitro blue tetrazolium detected at 560 nm. Catalase (CAT, EC 1.11.1.6) activity was determined by monitoring the decrease in absorbance at 240 nm due to the decomposition of H_2_O_2_ [46]. The peroxidase (POD; EC 1.11.1.7) activity was determined by analyzing the guaiacol oxidation at 470 nm according to the method used by [47]. Polyphenol oxidase (PPO, EC 1.10.3.1) activity was determined at 490 nm following the method described by [48]. Lipoxygenase (LOX, EC 1.13.11.12) activity was assayed at 234 nm using linoleic acid as a substrate solution as described by [49].

### 4.10. Statistical Analysis

A completely randomized design was used, with four replicates per treatment and with each replicate including six plants gathered from the same pot. A combined analysis was performed for the two growing seasons, since the results of the two seasons followed a similar trend. Data were analyzed by two-way ANOVA test, whereby the first factor was the salt treatments and the second was the foliar application treatments. Differences between the treatments were tested by least significant difference (LSD) test at a level of significance of *p* < 0.05. The data are presented as means ± standard errors (SE).

## 5. Conclusions

The present study reveals that salicylic acid co-applied with melatonin appears to be a great candidate for boosting wheat growth by mitigating salt toxicity. The salicylic acid–melatonin interaction improved the H^+^-pump activity and ROS detoxification, which in turn maintained the ionic homeostasis under saline conditions. These results show that the higher root H^+^-pump activity in treated plants is a tolerance mechanism that increases the salt tolerance via maintaining ionic homeostasis. These findings provide novel insight into the synergistic effects of salicylic acid and melatonin against salt stress that occurs through upregulating H^+^-ATPase activity; restricting Na^+^ absorption; improving N, P, K^+^, Fe, Zn, and Cu acquisition; maintaining K^+^/Na^+^, Ca^2+^/Na^+^, and Mg^2+^/Na^+^ ratios;increasing water content and osmolytes synthesis; alleviating salt-stress-induced oxidative damage; suppressing superoxide radicals production; stabilizing membranes; decreasing solute leakage; and enhancing antioxidant enzyme activity. Therefore, co-application of salicylic acid and melatonin as an environmentally friendly approach can serve as a potent elicitor against harsh environmental conditions in agronomic and horticultural crops.

## Figures and Tables

**Table 1:** Influence of salicylic acid (SA, 75 mg L^−1^), melatonin (MT, 70 μM), and SA (75 mg L^−1^) + MT (70 μM) foliar application treatments on the total leaf area plant^−1^, shoot dry weight plant^−1^, root dry weight plant^−1^, number of grains plant^−1^, and grain yield plant^−1^ of wheat plants grown under 0.1, 6, and 12 dS m^−1^ salinity levels.

Salinity Level EC (dS m^−1^) + Foliar Applications	Total Leaf Area Plant^−1^ (cm^2^)	Shoot Dry Weight Plant^−1^ (g)	Root Dry Weight Plant^−1^ (g)	Grain Number Plant^−1^	Grain Yield Plant^−1^ (g)
0.1	112.0 ± 3.2 ^e,f^	5.8 ± 0.15 ^e,f^	0.91 ± 0.07 ^c^	170.0 ± 3.3 ^e^	4.98 ± 0.17 ^d,e^
0.1 + SA	137.7 ± 3.4 ^c^	7.2 ± 0.14 ^c^	1.18 ± 0.09 ^b^	209.8 ± 3.7 ^c^	6.33 ± 0.24 ^b,c^
0.1 + MT	149.3 ± 3.3 ^b^	7.9 ± 0.16 ^b^	1.27 ± 0.11 ^b^	231.5 ± 3.6 ^b^	6.88 ± 0.26 ^b^
0.1 + SA + MT	166.7 ± 4.4 ^a^	8.9 ± 0.28 ^a^	1.50 ± 0.15 ^a^	257.1 ± 4.1 ^a^	7.83 ± 0.18 ^a^
6.0	89.0 ± 2.5 ^g^	4.5 ± 0.15 ^g^	0.66 ± 0.05 ^e^	130.3 ± 2.5 ^g^	3.09 ± 0.15 ^g^
6.0 + SA	117.3 ± 3.9 ^d,e^	6.0 ± 0.17 ^e^	0.92 ± 0.07 ^c^	176.5 ± 2.7 ^e^	4.38 ± 0.17 ^ef^
6.0 + MT	125.1 ± 2.8 ^d^	6.5 ± 0.13 ^d^	0.98 ± 0.13 ^c^	189.4 ± 3.3 ^d^	4.47 ± 0.13 ^e,f^
6.0 + SA + MT	143.5 ± 3.5 ^b,c^	7.8 ± 0.29 ^b^	1.19 ± 0.09 ^b^	228.6 ± 3.9 ^b^	5.68 ± 0.19 ^c,d^
12.0	60.0 ± 1.2 ^h^	2.6 ± 0.11 ^h^	0.40 ± 0.03 ^f^	80.1 ± 1.1 ^h^	2.11 ± 0.11 ^h^
12.0 + SA	88.0 ± 1.8 ^g^	4.4 ± 0.17 ^g^	0.68 ± 0.05 ^e^	132.0 ± 2.7 ^f,g^	3.45 ± 0.11 ^g^
12.0 + MT	94.0 ± 1.4 ^g^	4.7 ± 0.13 ^g^	0.73 ± 0.03 ^d,e^	143.6 ± 2.3 ^f^	3.79 ± 0.13 ^f,g^
12.0 + SA + MT	108.0 ± 2.9 ^f^	5.4 ± 0.19 ^f^	0.86 ± 0.07 ^c,d^	168.9 ± 2.9 ^e^	4.87 ± 0.19 ^e^

Means ± SE (*n* = 4) with different letters within the same column are statistically different according to LSD test (*p* < 0.05).

**Table 2:** Influence of salicylic acid (SA, 75 mg L^−1^), melatonin (MT, 70 μM), and SA (75 mg L^−1^) + MT (70 μM) foliar application treatments on the relative water content (%),total free amino acids concentration, and superoxide (O_2_^•−^) content levels in leaves of wheat plants grown under 0.1, 6, and 12 dS m^−1^ salinity levels.

Salinity Levels EC (dS m^−1^) + Foliar Applications	Relative Water Content (%)	Total Free Amino Acids Concentration (mg g^−1^ DW)	Superoxide (O_2_^•−^) Content (Δ A_580_g^−1^ DW)
0.1	66 ± 0.70 ^b,c^	5.1 ± 0.15 ^g^	28 ± 0.41 ^d,e^
0.1 + SA	68 ± 0.85 ^b^	5.4 ± 0.14 ^g^	27 ± 0.50 ^e,f^
0.1 + MT	70 ± 0.74 ^b^	5.4 ± 0.13 ^g^	27 ± 0.39 ^e,f^
0.1 + SA + MT	75 ± 0.87 ^a^	5.8 ± 0.14 ^f,g^	25 ± 0.38 ^f^
6.0	50 ± 0.65 ^f^	7.6 ± 0.17 ^e,f^	35 ± 0.56 ^b^
6.0 + SA	59 ± 0.91 ^d,e^	8.9 ± 0.20 ^d,e^	31 ± 0.37 ^c^
6.0 + MT	58 ± 0.73 ^e^	9.5 ± 0.22 ^d,e^	31 ± 0.43 ^c^
6.0 + SA + MT	69 ± 0.65 ^b^	10.8 ± 0.25 ^c,d^	26 ± 0.39 ^e,f^
12.0	39 ± 0.58 ^g^	9.8 ± 0.21 ^d^	44 ± 0.67 ^a^
12.0 + SA	53 ± 0.73 ^f^	12.3 ± 0.23 ^b,c^	37 ± 0.57 ^b^
12.0 + MT	49 ± 0.65 ^f^	14.0 ± 0.26 ^a,b^	35 ± 0.49 ^b^
12.0 + SA + MT	63 ± 0.97 ^c,d^	16.0 ± 0.22 ^a^	30 ± 0.39 ^c,d^

Means ± SE (*n* = 4) with different letters within the same column are statistically different according to LSD test (*p* < 0.05).

## Data Availability

The data presented in this study are available in the article and Supplementary Materials.

## Supplementary Materials

The following are available online at https://www.mdpi.com/article/10.3390/plants11030416/s1: Table S1: *p*-values of the two-way analysis of the measured parameters in wheat (*Triticum aestivum* L. cv. Sids 14) plants as affected by foliar treatments under different salinity levels. *P*-values in bold are considered significant (<0.05, n = 4). ‘S’: effect of salinity levels; ‘T’: effect of foliar treatments; S x T: effect of the variables’ interaction. Table S2: Chemical properties of the soil under different salinity levels.
