# Supplementary material for: Synergistic Effects of Salicylic Acid and Melatonin on Modulating Ion Homeostasis in Salt-Stressed Wheat (Triticum aestivum L.) Plants by Enhancing Root H+-Pump Activity
Source: Plants (Basel). 2022 Feb 2;11(3):416. doi: 10.3390/plants11030416 (PMC8840481; doi:10.3390/plants11030416)
Supplement: Supplementary file 1 [file plants-11-00416-s001.zip › plants-1505411-supplementary.pdf]

Table S1. *P*-values of the two-way analysis of the measured parameters in wheat (*Triticum aestivum* L. cv. Sids 14) plants as affected by foliar' treatments under different salinity levels. *P*-values in bold are considered significant (<0.05, n = 4). 'S': effect of salinity levels; 'T': effect of foliar treatments; S x T: effect of the variables' interaction.

		Main-factor effects		Significant interaction
		S	T	S x T
**Total leaf area plant^{-1}**		<0.0001	<0.0001	-
**Shoot dry weight plant^{-1}**		<0.0001	<0.0001	0.2488
**Root dry weight plant^{-1}**		<0.0001	<0.0001	-
**Grains number plant^{-1}**		<0.0001	<0.0001	0.3903
**Grain yield plant^{-1}**		<0.0001	<0.0001	-
**N concentration**	**shoots**	<0.0001	<0.0001	0.3886
	**grains**	<0.0001	<0.0001	-
**P concentration**	**shoots**	<0.0001	<0.0001	0.0076
	**grains**	<0.0001	<0.0001	0.3777
**K^+ concentration**	**shoots**	<0.0001	<0.0001	0.0042
	**grains**	<0.0001	<0.0001	-
**Na^+ concentration**	**shoots**	<0.0001	<0.0001	<0.0001
	**grains**	0.0001	0.0001	0.1417
**Ca^{2+} concentration**	**shoots**	<0.0001	<0.0001	0.0815
	**grains**	<0.0001	0.0004	-
**Mg^{2+} concentration**	**shoots**	<0.0001	<0.0001	0.0574
	**grains**	<0.0001	<0.0001	0.0351
**Fe concentration**	**shoots**	<0.0001	<0.0001	0.0627
	**grains**	<0.0001	<0.0001	0.0133
**Zn concentration**	**shoots**	<0.0001	<0.0001	0.1949
	**grains**	<0.0001	<0.0001	0.1228
**Cu concentration**	**shoots**	<0.0001	<0.0001	0.1268
	**grains**	<0.0001	<0.0001	0.0010
**K^+/Na^+ ratio**	**shoots**	<0.0001	<0.0001	0.0001
	**grains**	<0.0001	<0.0001	-
**Ca^{2+}/Na^+ ratio**	**shoots**	0.0003	<0.0001	<0.0001
	**grains**	0.0447	<0.0001	0.1209
**Mg^{2+}/Na^+ ratio**	**shoots**	0.3091	<0.0001	<0.0001
	**grains**	0.3277	<0.0001	0.0188
**ATP content**		<0.0001	<0.0001	0.0076
**PM H^+-ATPase activity**		<0.0001	<0.0001	0.0377
**VM H^+-ATPase activity**		<0.0001	<0.0001	0.0098
**VM H^+-PPase activity**		<0.0001	<0.0001	0.0081
**Relative water content %**		<0.0001	<0.0001	0.0009
**Free amino acids concentration**		<0.0001	0.0001	0.0340
**Superoxide content**		<0.0001	<0.0001	<0.0001
**Electrolyte leakage %**		<0.0001	<0.0001	0.0005
**Membrane stability index %**		<0.0001	<0.0001	0.0009
**Lipoxygenase activity**		<0.0001	<0.0001	<0.0001
**Superoxide dismutase activity**		<0.0001	<0.0001	<0.0001
**Catalase activity**		<0.0001	<0.0001	<0.0001
**Peroxidase activity**		<0.0001	<0.0001	<0.0001
**Polyphenol oxidase activity**		<0.0001	<0.0001	<0.0001

Table S2. Chemical properties of the soil under different salinity levels.

Salinity levels EC (dS m^{-1})	pH	HCO_3^- + CO_3^{2-} (mg kg^{-1})	Cl^- (mg kg^{-1})	SO_4^{2-} (mg kg^{-1})	Ca^{2+} (mg kg^{-1})	Mg^{2+} (mg kg^{-1})	Na^+ (mg kg^{-1})	K^+ (mg kg^{-1})
**0.1**	7.2	213.5	324.0	430.7	92.2	41.4	3.7	31.4
**6.0**	7.5	263.6	1173.4	996.9	398.5	173.9	306.7	39.7
**12.0**	7.8	275.4	1987.8	1686.1	886.5	314.5	808.6	52.6
